# Integrated nano-opto-electro-mechanical sensor for spectrometry and nanometrology

**DOI:** 10.1038/s41467-017-02392-5

**Published:** 2017-12-20

**Authors:** Žarko Zobenica, Rob W. van der Heijden, Maurangelo Petruzzella, Francesco Pagliano, Rick Leijssen, Tian Xia, Leonardo Midolo, Michele Cotrufo, YongJin Cho, Frank W. M. van Otten, Ewold Verhagen, Andrea Fiore

**Affiliations:** 1 Department of Applied Physics and Institute for Photonic Integration, Eindhoven University of Technology, P.O. Box 513, 5600 MB Eindhoven, The Netherlands; 2 Center for Nanophotonics, FOM Institute AMOLF, Science Park 104, 1098 XG Amsterdam, The Netherlands; 3 Niels Bohr Institute, University of Copenhagen, Blegdamsvej 17, 2100 Copenhagen, Denmark

## Abstract

Spectrometry is widely used for the characterization of materials, tissues, and gases, and the need for size and cost scaling is driving the development of mini and microspectrometers. While nanophotonic devices provide narrowband filtering that can be used for spectrometry, their practical application has been hampered by the difficulty of integrating tuning and read-out structures. Here, a nano-opto-electro-mechanical system is presented where the three functionalities of transduction, actuation, and detection are integrated, resulting in a high-resolution spectrometer with a micrometer-scale footprint. The system consists of an electromechanically tunable double-membrane photonic crystal cavity with an integrated quantum dot photodiode. Using this structure, we demonstrate a resonance modulation spectroscopy technique that provides subpicometer wavelength resolution. We show its application in the measurement of narrow gas absorption lines and in the interrogation of fiber Bragg gratings. We also explore its operation as displacement-to-photocurrent transducer, demonstrating optomechanical displacement sensing with integrated photocurrent read-out.

## Abstract (web summary)

Fully integratable spectrometers have trade-offs between size and resolution. Here, the authors present a nano-opto-electro-mechanical system where the functionalities of transduction, actuation and detection are fully integrated, resulting in an ultra-compact high-resolution spectrometer with a micrometer-scale footprint.

## Introduction

The increasing demand for optical sensing solutions, including e.g., Raman, fluorescence, and absorption spectroscopy, is driving a large effort toward the miniaturization and integration of spectrometers, which has resulted in a range of mini and microspectrometers^1–9^. Most schemes employ diffraction for the spectral discrimination, which inevitably brings a trade-off between size and resolution. Integrated spectrometer implementations^10–15^ are mostly based on arrays of filter elements, which limit the resolution, and rely on external detectors, resulting in a much increased packaging complexity and cost. In principle, the combination of a tuneable optical cavity and a photodetector can lead to an extremely compact spectrometer, particularly if the detector is integrated inside the cavity^16,17^. However, for many applications, high resolution is needed under a wide range of incident angles and over a wide spectral range. This can only be achieved by a wavelength-scale cavity combining low optical loss, wide free-spectral range (FSR), and large tuneability. So far, tuneable microcavity detectors have achieved limited resolution^17^ and spectral range^16^. Nano-optomechanical structures, such as photonic crystal (PhC) cavities^18,19^ and micro-ring resonators^20–23^, combine high spectral resolution and large optomechanical coupling, resulting in exquisite sensitivity to nanoscale mechanical motion^24^. This interaction between optical and mechanical degrees of freedom can be used to transduce pm-scale mechanical displacements into wavelength shifts and vice versa. It potentially opens the way to a new generation of ultra-compact optical sensors, particularly spectrometers, if the required control and read-out can be integrated with the sensing part. The device presented in this paper integrates the tuning, sensing, and read-out within a footprint of only 15 × 15 μm^2^ and provides high-resolution spectra even under a large numerical aperture (NA) illumination, when operated as a tunable filter (spectrometer). We further show that wavemeter measurements of a single laser line or an absorption dip in a broad background are possible with a precision three orders of magnitude better than the optical cavity linewidth. Finally, we demonstrate displacement sensing in the same device, using a fixed laser line as input and integrated detection to transduce the thermal motion of the structure.

## Results

### Device design

**Fig. 1:**
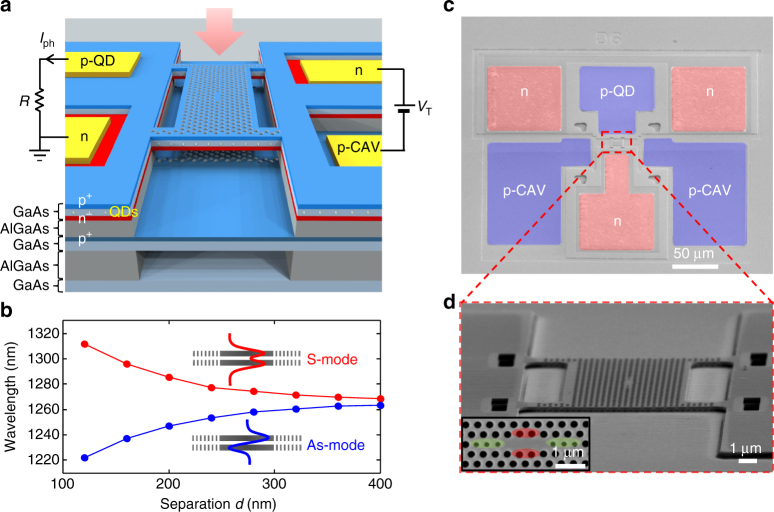
Overview of the sensor design. **a** Sketch of the device with electrical contacts and a visible cross-section with p- (blue) and n- (red) doped layers. QDs are located in the middle of the top membrane. Sensor actuation is possible by applying a reverse bias voltage (*V*_T_) to the tuning diode (on the right side), whereas the read-out is done by measuring the photocurrent from the photodiode (left side). **b**, Simulated optical mode wavelength dependence on the membrane separation for two modes that are symmetric (S) or antisymmetric (As) with respect to the out-of-plane direction. **c** False-colored SEM image of a typical device (top view) with contact pads to both sensing and actuation diodes. **d** Zoom-in SEM image showing the active part of the sensor: a four-arm bridge of dimensions 16 × 12 μm containing a photonic crystal cavity suspended above a fixed photonic crystal membrane. Inset: SEM image of the patterned L3 cavity design modified for high *Q* factor and large free-spectral range in a double-membrane structure. Optimization was done by displacing horizontally outwards and reducing the radius of six holes (green) and displacing four holes vertically (red)

Figure 1 shows the nano-opto-electro-mechanical system (NOEMS) we employ. Our approach (Fig. 1a) is based on an electromechanically tuneable, double-membrane PhCs^25–27^, and a low-absorption active material (quantum dots (QDs)). Two identical cavities in the two parallel membranes are evanescently coupled so that the two degenerate cavity modes split into combined symmetric (S) and an antisymmetric (As) modes. The resonant wavelengths strongly depend on the separation *d* between the membranes, as shown in the simulated tuning curves of Fig. 1b. In the range of *d*~200 nm, the optical angular frequency shift per displacement *G*_*ω*_ = *∂ω/∂x* is in the range of 2*π* × 37 GHz nm^−1^ (*∂λ/∂x* = 0.2 nm per nm)^27^. The lower part of the upper membrane and the upper part of the lower membrane are doped in order to form a p-i-n diode. The distance between the membranes can be controlled by using electrostatic actuation provided by a reverse bias voltage *V*_T_ across the p-i-n diode. Compared to in-plane capacitive tuning^28–30^, vertical-actuation offers larger capacitance, resulting in more efficient actuation and smaller footprint. Moreover, it enables the actuation and sensing of out-of-plane motion that is relevant for most nanometrology applications, such as atomic force microscopy. The upper membrane is configured as another p-i-n diode, the n-layer being common to both diodes, see Fig. 1a. A layer of InAs QDs, absorbing in the resonant wavelength range, is grown at the center of the upper membrane. The modal absorption, and thereby the detector efficiency and the cavity loss, can be adjusted by controlling the density of the QDs. We chose the dot density so that the absorption contribution to the cavity loss does not limit the *Q* factor. We estimate the modal absorption^31^ to be *α*_mod_ = 2.41 cm^−1^, corresponding to an absorption-limited quality factor (*Q*_abs_) of 6.8 × 10^4^. The PhC cavities are modified L3 or H0 cavities where the position and size of the holes close to the cavity center have been optimized to achieve at the same time a high quality factor and a wide spectral separation (Methods).

### Resonant detection

**Fig. 2:**
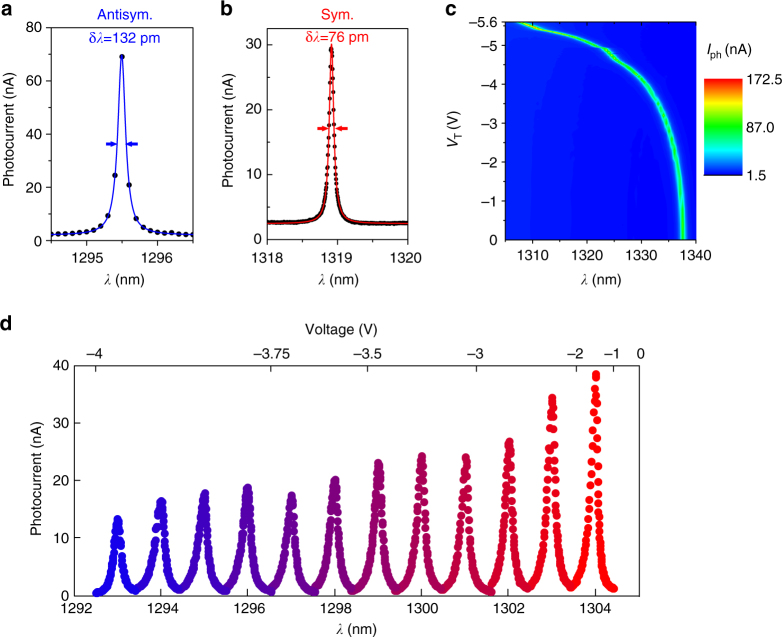
μ-spectrometer measurements. **a**, **b** A photocurrent spectrum of the fundamental antisymmetric (symmetric) mode of the three missing holes cavity (L3) modified for high *Q* factor in double membrane, with a linewidth of 132 pm (76 pm) and a *Q* factor of *Q*_As_~9900 (*Q*_S_~17,000) is shown in blue (red). Data was taken by measuring the photocurrent while a tuneable laser (*P*_in_ = 125 μW) was swept across the cavity mode. **c** Color-coded photocurrent spectra (*P*_in_ = 25 μW) showing the fundamental antisymmetric optical modes of an optimized H0 cavity spectrally tuned over 30 nm (*x* axis) by increasing the reverse tuning bias *V*_T_ from 0 to 5.6 V (*y* axis) without reaching pull-in. The tuning range is approximately equal to one free spectral range in this case. **d** Data traces of photocurrent collected by voltage-tuning the optical mode (fundamental antisymmetric mode of the L3 cavity) over a fixed laser wavelength, then changing the laser wavelength by 1 nm and continuing the voltage tuning. The laser power was coupled into the cavity from top through a 0.45 NA objective, with the power incident on the sample being 12.5 μW. The scale on the bottom axis in the figure is obtained from a piecewise linear fit of the voltages at the maximum photocurrent versus wavelength. The cavity linewidth provides a spectral resolution of ~200 pm, and the FSR of ~13 nm is limited by the crossing with another cavity mode. The decrease in responsivity with decreasing inter-membrane distance (decreasing wavelength) is attributed to an asymmetry in membrane thickness, the upper membrane being 15 nm thicker^46^

To demonstrate the resonant detection functionality, light from a tuneable laser is coupled into the cavity from the top with a fixed bias on the actuation junction (Fig. 2a, b for the antisymmetric and the symmetric mode, respectively). The photocurrent spectrum shows the cavity resonance, apart from a non-resonant background, and is a result of cavity-enhanced absorption. An experimental cavity linewidth for a symmetric fundamental PhC mode as narrow as 76 pm (*Q*_S_ = 1.7 × 10^4^) was obtained utilizing the optimized cavity design from Fig. 1d (inset), while corresponding antisymmetric mode is slightly wider, with a linewidth of 132 pm (*Q*_As_ = 9.9 × 10^3^). Such narrow linewidth corresponds to an order of magnitude improvement over previous reports in resonant cavity detectors^17^. We further investigate the tuneability. As for this particular design, the symmetric cavity mode is located close to the antisymmetric band edge, with several nearby peaks, we consider the antisymmetric mode in the following. As shown in Fig. 2c, blue-tuning of such mode by as much as 30 nm is obtained for a small applied voltage of 5.6 V, corresponding well to the simulated membrane tuning until the pull-in limit (1/3 of the original distance) inherent to capacitive tuning^32^. The tuning range can be extended to 40–50 nm if the initial membrane separation in the design is reduced by a factor of two (from 240 to 120 nm), and even further if actuation beyond pull-in is realized^33^. The mode used in Fig. 2c is the fundamental antisymmetric mode of a H0 cavity optimized for high FSR, where we define FSR as the maximum wavelength range for which there is only the mode of interest. Large FSR in this case comes at a price of a larger linewidth of 0.7 nm (*Q*_exp_ = 1.9 × 10^3^). The device maps the combination of the incident spectral power density *S*(*ω*) and inter-membrane distance *d* into a photocurrent signal $I_\varphi \left( d \right) = R\mathop {\int}\nolimits_{ - \infty }^\infty {S\left( \omega \right)} L_{{\mathrm{cav}}}\left( {d,\omega } \right)\mathrm{d}\omega$, where *R* is the responsivity (A W^−1^) and *L*_cav_ (*d*, *ω*) the normalized spectral shape of the cavity resonance, which is centered at frequency *ω*_0_ (*d*). It can therefore be operated to sense either the spectrum of the incident radiation or the mechanical displacement by recording the photocurrent. In the spectrometer mode, the input spectral power density *S*(*ω*) is measured by actuating the membrane separation, *d* = *d*(*V*_T_), and for displacement sensing, the membrane separation *d* can be deduced from the resonance frequency. The spectrometer operation is demonstrated for a cavity mode where both *Q* and FSR are sufficiently high (Fig. 2d), which is the case for the fundamental antisymmetric mode (Y1-As) of the modified L3 cavity, with a calculated *Q* = 1.2 × 10^4^ and FSR = 13 nm. For a number of fixed laser frequencies, a voltage sweep is made across the resonances. Peak positions were taken as calibration points, with which the voltage scale (top) was converted to the wavelength scale (bottom) in Fig. 2d.

The peak photodiode responsivity for the data in Fig. 2d is *R* *~*3 × 10^−3^ A W^−1^ (Methods). It is limited by the small absorptance (*η*_a_ = 0.1), which can be increased by increasing the dot density without a large influence on the *Q*, as well as unoptimized coupling efficiency (*η*_c_ = 0.03) that can be improved using a side-coupling scheme. The cavity photocurrent peak (Fig. 2b) is superimposed on a non-resonant background caused by light that is directly absorbed in the top membrane. The limited stray light rejection ratio (typically 10–20 dB), may be detrimental when a small spectral feature must be measured on a broad background.

### Resonance modulation spectroscopy

**Fig. 3:**
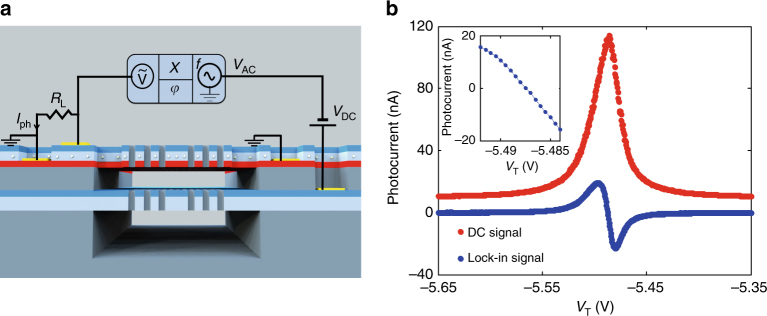
Resonance modulation spectroscopy. **a** Sketch of the circuit used for resonance modulation spectroscopy. On the tuning probe input (right side), a modulated signal (*V*_AC_) at frequency *f* coming from the lock-in is superimposed on a tuneable DC voltage (*V*_DC_). On the detector output (left side), the photocurrent (*I*_ph_) produces a voltage drop on a load resistor (*R*_L_) and its in-phase component (*X*-channel) and its phase (*φ*) at the frequency (*f*) are measured by the lock-in amplifier. **b** Comparison of a laser line recorded using two operation modes of the sensor: spectrometer mode described earlier (red dots) and resonance modulation mode (blue dots). Both measurements were performed simultaneously, by sweeping the tuning voltage and reading the photocurrent DC value (red) and the in-phase component measured by the lock-in amplifier (blue), using a load resistor *R*_L_ = 30 kΩ. Since the blue curve is proportional to the derivative of the red curve, the constant background is eliminated. The cavity is modulated with *V*_AC_ = 5 mV_pp_ at *f* = 608 Hz, and excited at a fixed laser frequency of 1322 nm (the power incident on the sample is estimated to be 125 μW); Inset: zoom-in of the zero crossing in the resonance modulation mode

We introduce a resonance modulation spectroscopy scheme, which can at the same time suppress the effect of background absorption and dramatically increase the spectral peak position resolution. It is based on the small size and built-in actuation functionality of our NOEMS, which enables modulating the mode resonant wavelength at frequencies up to the MHz range. Applying a small modulation to the tuning voltage as $V_{\mathrm{T}} = V_{{\mathrm{DC}}} + V_{{\mathrm{AC}}}\,{\rm cos}\left( {2\pi f_{\mathrm{m}}t} \right)$, the cavity frequency *ω*_cav_ is modulated around its central value *ω*_0_ (*V*_DC_) and the photocurrent ${{\delta I}}_{{\varphi }}^{{f}_{\rm m}}$ at frequency *f*_m_, as measured using a lock-in amplifier (Fig. 3a), becomes:

1 $$\delta I_\varphi ^{f_{\mathrm{m}}}\left( {\omega _0\left( {d_0} \right)} \right) = R\,\delta d\,\mathop {\int}\limits_{ - \infty }^\infty {\frac{{\partial L_{{\mathrm{cav}}}\left( {d,\omega } \right)}}{{\partial d}}} S\left( \omega \right)\mathrm{d}\omega \\ = R\,\delta \omega _{\mathrm{m}}\,\mathop {\int}\limits_{ - \infty }^\infty {\frac{{\partial L_{{\mathrm{cav}}}\left( {\omega _0,\omega } \right)}}{{\partial \omega _0}}} S\left( \omega \right)\mathrm{d}\omega ,$$

where ${\mathrm{\delta \omega }}_m = {\mathrm{G}}_{\mathrm{\omega }}{{\delta d}}$ is the frequency modulation depth (which we assume much smaller than the optical linewidth). Note that *L*_cav_ is assumed to be a Lorentzian $L_{\mathrm{cav}} ({\omega} - {\omega}_{0})$ of constant width, so that $\partial L_{{\mathrm{cav}}}{\mathrm{/}}\partial \omega _0 = - \partial L_{{\mathrm{cav}}}{\mathrm{/}}\partial \omega$. In the limit where *S*(*ω*) is much narrower than the cavity linewidth, $\delta I_\varphi ^{f_{\mathrm{m}}}\left( {\omega _0} \right)$ is proportional to the derivative of the cavity resonance lineshape. In the opposite limit of a slowly varying input spectrum, $\delta I_\varphi ^{f_{\mathrm{m}}}\left( {\omega _0} \right)$ is proportional to the derivative of the input spectrum $\left. {\frac{{\mathrm{d}S}}{{\mathrm{d}\omega }}} \right|_{\omega _0}$ as immediately follows from integrating Eq. (1) by parts. The output signal therefore exclusively results from spectral features at the mode frequency and any spectrally flat background is rejected. The principle is demonstrated experimentally for a narrow laser line in Fig. 3b, showing a large improvement of the rejection ratio, from 10 to 27 dB, with values up to 30 dB measured in other devices. The sign-changing lineshape of the ac photocurrent amplitude also lends itself to the generation of an error signal for feedback-based stabilization. Similarly to frequency^34^ and wavelength^35^ modulation methods, the resonance modulation scheme allows measuring the position of spectral lines with resolution much better than the linewidth—but it can be applied much more widely as it does not require a modulation of the source. From the slope of the derivative curve at the zero crossing (inset Fig. 3b), the voltage–wavelength relation and the measured noise, we calculate a spectral resolution of 100 fm Hz^−1/2^ (Methods), limited by the drift of the cavity resonance during the measurement time. This long-term drift, which produces resonant wavelength shifts in the pm-range over timescales of tens of seconds, is likely related to the adsorption of residual gases on the surface and in the holes of the PhC and temperature drifts. The intrinsic resolution, as limited by the electrical noise in the read-out, is estimated to be ∼2 fm Hz^−1/2^.

**Fig. 4:**
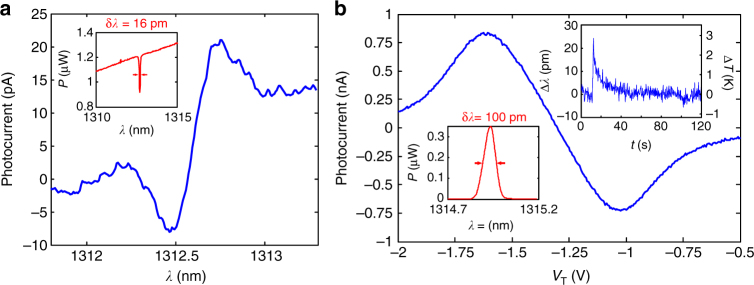
Applications of resonance modulation spectroscopy. **a** Gas sensing demonstration, where an HF absorption line at 1312.591 nm is measured in transmission using the resonance modulation scheme previously described. Voltage was translated into wavelength using a calibration curve obtained from independent PL cavity mode tuning data. A superluminescent diode (SLED) fiber-coupled to an HF gas cell (at *p* = 50 Torr), and filtered with a 12 nm wide 1310 nm band pass filter is used for excitation. A filter is needed to isolate a single absorption line, and a single cavity mode; inset: optical spectrum analyzer (OSA) spectrum of the SLED with the HF cell inserted, showing the same absorption line. **b** Wavemeter measurement of a FBG resonance in reflection performed using the resonance modulation scheme. A SLED is connected to the first input of a 2 × 2 fiber beam-splitter with the FBG on one of the outputs, and the second input (reflection) is coupled to the cavity through a NA = 0.45 objective (*P*_in_ ≈ 1.6 μW). The FBG peak (100 pm wide) is read by sweeping a cavity mode (*δλ*_c_ = 237 pm, *δV*_c_ = 270 mV), having a sensitivity (slope at zero crossing) of *S*_*I*_ = 3.53 nA V^−1^. The noise measured at the zero crossing is *δI*_noise_ = 3.6 pA Hz^−1/2^ that translates to a peak wavelength uncertainty of *δλ*_noise_ = (*δI*_noise_/*S*_*I*_) × (*δλ*_c_ */δV*_c_) = 0.9 pm Hz^−1/2^. Inset left: the OSA spectrum of the FBG filter in reflection. Inset right: detuning of the FBG peak (left axis) and corresponding temperature shift (right axis) over a period of 120 s measured using the cavity sensor. The peak visible at *t* = 10 s is induced by convective heating from a heat-gun 50 cm away from the FBG. The current signal (lock-in output) is translated to displacement (∆*λ*) using the slope *S*_*I*_. The FBG temperature sensitivity is taken to be *δλ*_B_ */δT* = 8.5 pm K^−1^ (specified by the manufacturer)

Despite the relatively limited spectral range (up to 30 nm with the present devices, Fig. 2c), these very compact microspectrometers can be employed to monitor specific spectral lines for sensing applications, with outstanding resolution. In order to show their potential in real-world applications, two experiments requiring a high-resolution spectral measurement were conducted: the read-out of a narrow absorption line in transmission (Fig. 4a), and the interrogation of a fiber Bragg grating (FBG) in reflection (Fig. 4b). For the measurement of the absorption line in a broad spectrum, the background rejection provided by the resonance modulation scheme is essential. The experiment is demonstrated in Fig. 4a, where a hydrofluoric acid absorption line is detected, despite the fact that its linewidth (16 pm) is about 15 times narrower than the cavity linewidth used in this experiment (250 pm). The high peak position resolving power of our device also makes it useful for the read-out of temperature, index or pressure sensors based on spectral peak position determination. An example of a FBG peak (*λ*_B_ = 1314.823 nm, *δλ*_B_ = 100 pm) read-out in reflection is presented in Fig. 4b, with a wavelength resolution (as calculated from the lock-in noise) of 0.9 pm Hz^−1/2^_._ Without changing the tuning voltage (*V*_T_), a range of ~500 pm can be covered, and with the tuning it can be extended to ~20 nm. Both resolution and spectral range are comparable to commercial implementations^36^, but they are here obtained with a much smaller footprint (optically active part 15 × 15 μm compared to approximately cm scale device). The peak position was monitored in time with the NOEMS sensor, and small detunings ~1 pm induced by a short pulse of convective heating (∆*T*~ 0.1 K) were succesfully detected (right inset in Fig. 4b). We note that our spectrometer enables the read-out of sensors with very high resolution using a broad spectral source, releasing the need for expensive tuneable lasers. The current implementation can be extended to read a multiplexed input with the use of multiple cavities connected by on-chip waveguides, for multispectral nanometrology applications.

### Displacement sensing

**Fig. 5:**
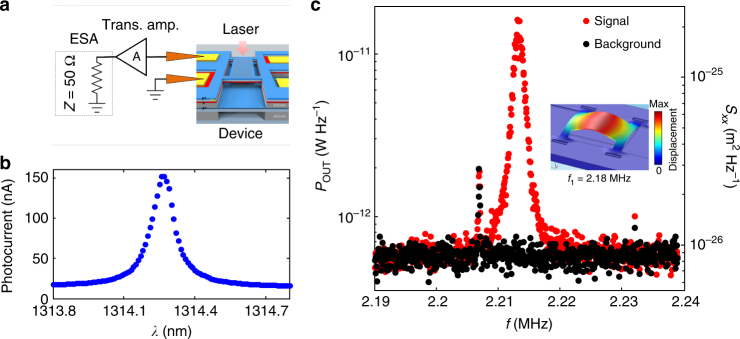
Brownian motion detected via photocurrent noise. **a** Sketch of the measurement circuit, with indicated device, input laser, RF probe, amplifier, and the ESA. An RF probe is used to measure the on-chip photodiode, and the signal is sent via an transimpedance amplifier (A in the setup sketch) to the input of the ESA. The device was mounted in a vacuum chamber (pressure below 10^−4^ mbar). In this experiment, no bias is supplied to the tuning diode. **b** Photocurrent measurement of the cavity optical mode for the laser input power *P*_in_ = 100 μW. **c** ESA spectrum of the photocurrent noise where the fundamental mechanical mode is visible in the output power (red dots) and control measurement with laser off (black dots). The right axis shows the calibrated power spectral density of motion. The CW laser was coupled into the cavity and the laser wavelength was red-detuned from resonance to the wavelength where the photocurrent varies maximally with detuning (*λ*_L_ = 1314.29 nm) and its power was kept low enough so as not to excite self-oscillations (*P*_in_ = 100 μW). The two other sharp features present in both measurements originate from the environmental RF noise; inset: 3D displacement plot of the 4-arm bridge fundamental mechanical mode with frequency *f*_1_ = 2.18 MHz simulated using Comsol

To demonstrate the motion sensor functionality, displacement fluctuations due to the Brownian thermal motion of the upper membrane were measured through the photocurrent, which directly monitors the intracavity field. A laser, red-detuned from a high-*Q* cavity mode (Fig. 5b), is coupled into the cavity and the photocurrent spectrum is measured by an electronic spectrum analyzer (ESA), see Fig. 5a. Transduced thermal motion of the fundamental flexural mode of the top membrane with a frequency *Ω*_M_ */*2*π* = 2.2 MHz and quality factor *Q*_M_ ≈ 1400 is observed, see Fig. 5c. The observed resonant fluctuations can be converted to a displacement spectral density *S*_*xx*_ (Fig. 5c, right axis) by equating them to the thermal variance <*x*_th_^2^> = *k*_B_ *T*/*m*_eff_ *Ω*_M_^2^, with *T* = 297 K and *m*_eff_ = 50 pg, obtained from finite element method (FEM) simulations (inset in Fig. 5c)^37^. The measurement imprecision is estimated to be 100 fm Hz^−1/2^ and is presently limited by the pick-up electrical noise in the measurement setup, which produces the noise floor in Fig. 5c. It could be improved to below 1 fm Hz^−1/2^ by optimizing the connection between device and amplifier and increasing the efficiency.

## Discussion

In summary, we have presented the concept of an integrated nanophotonic sensor that embodies the unique features of direct wavelength/displacement detection via photocurrent, and independent voltage control of the optical and mechanical properties of the structure via electrostatic actuation. We demonstrated a high-resolution microspectrometer and proved displacement sensing capabilities on a single device based on coupled PhC membranes. Furthermore, we introduce a resonance modulation spectroscopy method, exploiting the electromechanical control of the mode wavelength to reject stray light and increase the spectral resolution well beyond the cavity linewidth. Owing to the ultra-compact size (15 × 15 μm^2^) of the sensing element, this platform opens the way for mass production of multipurpose high-resolution sensors with embedded read-out. As examples of their potential, we present the measurement of a narrow (16 pm) gas absorption line and the interrogation of a FBG with sub-pm resolution. The sensor concept can be easily applied to other material systems to cover different wavelength ranges (from the visible to the mid-infrared), and further developed for application in temperature, refractive index, and electrical field sensing. The III–V semiconductor platform used for the device could be further exploited to integrate the light source, opening the way to fully integrated optical and optomechanical sensors requiring no external optical connections.

## Methods

### Sample structure

The sample was epitaxially grown by molecular beam epitaxy and consists of two GaAs slabs with nominal thicknesses of 170 nm (bottom) and 185 nm (top), separated by a 240 nm thick sacrificial Al_0.7_Ga_0.3_As layer. A 1.5 μm thick Al_0.7_Ga_0.3_As bottom sacrificial layer separates the membranes from the undoped (001) GaAs substrate. QDs (areal density 70 QDs per μm^2^, with ground-state absorption centered at 1310 nm at room temperature) are grown in the middle of the upper slab in a Stranski–Krastanov growth process. The upper 70-nm-thick part of both membranes was p-doped, while the bottom 70-nm-thick part of the top membrane was n-doped (*p*_upper_ = 1.5 × 10^18^ cm^−3^, *n* = *p*_lower_ = 2 × 10^18^ cm^−3^).

### Fabrication process and tolerances

The fabrication process begins with defining the vias for the contact pads for the two diodes of the device in two optical lithography steps followed by selective wet and dry etching steps to reach the bottom p- and the middle n-layer. In the p-via lithography step, four flexible arm bridges, which determine the stiffness of the top membrane, are also defined. To prevent the stress-induced buckling of the bridge, stress-release structures^38^ were implemented (Fig. 1d). No arms are etched in the lower membrane, making it mechanically much less compliant than the upper one. In the third optical lithography step, contact pads for all three doped layers are defined and metals are evaporated. After a lift-off step, 400 nm of Si_3_N_4_ is deposited on the sample (hard mask), ZEP resist is spun, and electron-beam lithography (EBL) at 30 kV is performed to define the PhC pattern. After development, the PhC pattern is transferred onto the hard mask using RIE (reactive ion etching) with CHF_3_. Resist is then removed with oxygen plasma, and the PhC pattern is imprinted as an array of holes in both membranes using a Cl_2-_based inductively coupled plasma (ICP) etching step. Release of the free-standing structure is done by selective wet etching of the sacrificial layer using a cold (1 °C) HCl solution. To prevent membrane stiction due to capillary forces, supercritical drying in CO_2_ is employed. Finally, the hard mask is removed by isotropic O_2_-CF_4_ plasma dry etching. The spectral position of the cavities was aligned to the peak of the QDs absorption spectrum by lithographic tuning of the PhC lattice constant. The QD inhomogeneous broadening (50 nm full width at half maximum in the present sample) could be further increased to reduce the impact of possible QD-cavity spectral misalignment and achieve a wide spectral photocurrent response. The cavity mode position is observed to fluctuate from device to device in a given process in a range of 5 nm due to various fabrication imperfections: buckling of the top membrane due to residual stress; alteration of the thickness of the top membrane during the dry and wet etchings steps (<10 nm); changes in the hole radii with respect to design (<10 nm) and roughness along the hole edges during the ICP etching of the holes; deviations in the lattice constant and position of the holes as determined by the precision of the EBL (<2 nm). Most of these issues lead to the spectral shift of the modes and/or to a change of the intensity of one of the two types of modes (S or As). The *Q* factor of the resonances is especially influenced by the ICP etching, hole shape, and roughness, making it the most critical step. We note that all these tolerances are related to the quality of the fabrication process, which could be substantially improved using industrial nanofabrication equipment.

### PhC cavity design

The light sensing double-membrane PhC cavity was designed to ensure small size (*V*  ∝ *λ* ^3^), high *Q*, and large FSR, by modifying standard L3 (with three holes missing in the hexagonal PhC^39^) and H0 (holes displaced around a position in the lattice^40^) designs. In the design of the L3 cavity used in the experiment in Figs. 2a, b, d, 3, 4 and 5, the position and the radii of the closest six holes in the *x*-direction (displacements *s*_1_/*a* = 0.3, *s*_2_/*a* = 0.225, *s*_3_/*a* = 0.1; radii *r*_1_ = *r*_2_ = *r*_3_ = 0.6 × *r*, with *r*/*a* *=* 0.306, the radius of the holes in the PhC and *a* the lattice parameter), and the position of the four holes in the *y*-direction (*h*_1_/*a* = 0.05) were optimized, providing a simulated *Q* factor of *Q*_cold_ = 4.4 × 10^4^ for the fundamental symmetric mode (Y1-S) in a cavity without the absorber and a mode spacing of ∆*λ* = 24 nm (from 3D FEM simulations). In the case of the fundamental symmetric mode (Y1-S), the experimental result mentioned in the main text (Fig. 2b), provides the *Q* factor of *Q*_exp_ = 1.7 × 10^4^. The absorption losses were estimated to give *Q*_abs_ ≈ 6.8 × 10^4^. We attribute the additional loss to scattering losses, patterning errors, and other fabrication imperfections: *Q*_fabr_ = (1/*Q*_exp_ − 1/*Q*_cold_ − 1*/Q*_abs_)^−1^ = 4.7 × 10^4^. The H0 cavity used for the experiment in Fig. 2c was designed for increased FSR by optimizing the position and radius of four holes. For a triangular lattice with *r*/*a* = 0.33 and parameters *s*_*x*_ = 0.15, *s*_*y*_ = 0.06, and radii, *r*_*x*_/*r* = 0.7 and *r*_*y*_/*r* = 0.75, a simulated mode spacing of ∆*λ* = 30 nm and *Q* factor of 1.8 × 10^4^ were obtained.

### Responsivity and coupling efficiency

The peak responsivity is given by *R* = (*e*/*hν*)*η*_c_ *η*_a_ *η*_i_ (*e* elementary charge, *h* Planck constant, *ν* light frequency, *η*_c_ coupling efficiency of light into the cavity mode, *η*_a_ fraction of cavity photons absorbed by the QDs, *η*_i_ internal efficiency of converting absorbed photons into collected electrons and holes). The internal efficiency is estimated to be close to 1, as we did not observe any change of photocurrent with applied reverse bias on the detector junction, indicating that carriers are efficiently extracted from the QDs. The current responsivity is limited by *η*_a_ and *η*_c_. The absorption and thereby *η*_a_, which is given by *η*_a_ = *Q*_exp_ */Q*_abs_, can be increased by increasing the QD density and number of QD layers, until the point when the absorption losses are comparable to the scattering losses, analogous to designs of Fabry Perot resonant cavity-enhanced photodetectors^41^. The free-space coupling currently employed is expected to have a low efficiency *η*_c_ (3% in the main text) due to the mismatch between the **k**-vector distribution of the incident field and the one of the cavity mode. The L3 design can be optimized to reach free-space coupling efficiencies up to 15% while maintaining a high *Q* ^42^, and with grating couplers cavity coupling efficiencies of 20% have been obtained^43^. It is known that coupling with on-chip waveguides can be very efficient (>50%^44,45^). Preliminary results of simulations show that similar coupling efficiencies can be expected when coupling waveguides to double-membrane cavities.

### Experimental setup

Light from a tuneable laser (Santec TSL-510) was coupled into the cavity from the top through a ×50 objective (NA = 0.45). All laser powers indicated in the main text are values incident on the sample. The two diodes were connected using two adjustable RF probes. In Figs. 2 and 3, the photocurrent was measured as a voltage drop on a 30 kΩ load resistor (R in Fig. 1a). For the measurements in Figs. 4 and 5, the photocurrent was amplified using a transimpedance amplifier (*A* = 5 × 10^5^ V A^−1^). All measurements were performed at room temperature. The thermal noise measurements in Fig. 5 were performed under vacuum conditions (*p* < 10^−4^ mbar) to suppress viscous air damping.

### Wavelength resolution in the resonance modulation scheme

The wavelength resolution in Fig. 3b is determined by measuring the current noise in the read-out when laser is on resonance with the cavity, *δI*_noise_ = 50 pA Hz^−1/2^ (measured using the lock-in-amplifier) and the slope of the derivative curve at the zero crossing *S*_*I*_ = 5 μA V^−1^, from which we calculate the voltage accuracy to be *δV*_T_ = *δI*_noise_/*S*_*I*_ = 10 μV Hz^−1/2^. With the mode wavelength tuning rate being 10 nm V^−1^, this voltage accuracy can be translated into to a (peak position) resolution of 100 fm when measured in 1 Hz bandwidth, a value 3 orders of magnitude smaller than the linewidth. As mentioned in the main text, this value is limited by the long-term drift of the cavity resonance. The fundamental noise limit, in the case where no drift is present, would be determined by the thermal noise of the load resistor (~0.7 pA Hz^−1/2^), corresponding to a potential wavelength resolution of ∼2 fm Hz^−1/2^, while the photon shot noise (~10 fA Hz^−1/2^) is negligible.

### Data availability

All relevant data is available from the corresponding author upon reasonable request.
